# Randomized Trial on the Effects of Dietary Potassium on Blood Pressure and Serum Potassium Levels in Adults with Chronic Kidney Disease

**DOI:** 10.3390/nu13082678

**Published:** 2021-07-31

**Authors:** Sharon Turban, Stephen P. Juraschek, Edgar R. Miller, Cheryl A. M. Anderson, Karen White, Jeanne Charleston, Lawrence J. Appel

**Affiliations:** 1 School of Medicine, Johns Hopkins University, Baltimore, MD 21087, USA; ermiller@jhmi.edu (E.R.M.III); kwhite33@jhmi.edu (K.W.); lappel@jhmi.edu (L.J.A.); 2 Beth Israel Deaconess Medical Center, Boston, MA 02215, USA; sjurasch@bidmc.harvard.edu; 3 Bloomberg School of Public Health, Johns Hopkins University, Baltimore, MD 21087, USA; jeannec@jhmi.edu; 4 The Welch Center for Prevention, Epidemiology and Clinical Research, Johns Hopkins University, Baltimore, MD 21087, USA; 5 Herbert Wertheim School of Public Health and Human Longevity Science, University of California at San Diego, San Diego, CA 92093, USA; c1anderson@ucsd.edu

**Keywords:** potassium, blood pressure, hypertension, chronic kidney disease, hyperkalemia

## Abstract

In the general population, an increased potassium (K) intake lowers blood pressure (BP). The effects of K have not been well-studied in individuals with chronic kidney disease (CKD). This randomized feeding trial with a 2-period crossover design compared the effects of diets containing 100 and 40 mmol K/day on BP in 29 adults with stage 3 CKD and treated or untreated systolic BP (SBP) 120–159 mmHg and diastolic BP (DBP) <100 mmHg. The primary outcome was 24 h ambulatory systolic BP. The higher-versus lower-K diet had no significant effect on 24 h SBP (−2.12 mm Hg; *p* = 0.16) and DBP (−0.70 mm Hg; *p* = 0.44). Corresponding differences in clinic BP were −4.21 mm Hg for SBP (*p* = 0.054) and −0.08 mm Hg for DBP (*p* = 0.94). On the higher-K diet, mean serum K increased by 0.21 mmol/L (*p* = 0.003) compared to the lower-K diet; two participants had confirmed hyperkalemia (serum K ≥ 5.5 mmol/L). In conclusion, a higher dietary intake of K did not lower 24 h SBP, while clinic SBP reduction was of borderline statistical significance. Additional trials are warranted to understand the health effects of increased K intake in individuals with CKD.

## 1. Introduction

Chronic kidney disease (CKD) increases the risk of cardiovascular disease (CVD) and premature death. Hypertension, which is common in the setting of CKD, likely contributes to the increased risk of CKD progression and development of CVD. Potassium (K) has been shown to decrease systolic BP (SBP) and diastolic BP (DBP) in persons with hypertension and, to a lesser extent, in persons without hypertension [1,2]. Evidence from animal studies as well as epidemiological studies in humans demonstrate that diets rich in K might reduce the risk of CVD [2]. These effects may be mediated by BP-dependent and BP-independent mechanisms.

The effects of K in individuals with CKD have not been well-studied, but animal and recent human studies have demonstrated that a high-K diet might lower BP and may protect against and/or reverse kidney damage [3,4,5,6,7,8,9]. However, intervention studies that test the effects of increased dietary K in persons with CKD are sparse, in part, because of concern about the occurrence of hyperkalemia in persons with CKD. Consequently, although diets high in K are recommended for the general population, patients with CKD are often advised to limit dietary K intake. In 2004, the National Kidney Foundation’s Kidney Disease Outcomes Quality Initiative (KDOQI) group recommended a K intake between 51 and 102 mmol/day (2 and 4 g/day, respectively) for individuals with stage 3–4 CKD [10]. Due to concerns about hyperkalemia, the 2004 KDOQI upper limit of 102 mmol/day was set below 120 mmol/day, the Adequate Intake level for the general population [11]. Renal K excretion, however, may not significantly decrease until glomerular filtration rate (GFR) is severely decreased (<10–20 mL/min/1.73 m^2^) [12,13,14]. Furthermore, individuals with moderate CKD may particularly benefit from higher rather than lower K intake, due to their higher prevalence of hypertension and higher risk of CVD, and because a higher K intake may slow the progression of CKD [7,15,16]. In 2019, Kidney Disease Improving Global Outcomes (KDIGO) highlighted the lack of evidence on K intake and CKD outcomes and concerns that routine dietary K restriction may limit patients from the benefits of K-rich foods [17].

Our objective was to conduct a controlled feeding trial, entitled CKD-K, to determine the health effects, including safety, of higher (100 mmol (3.9 g) K/day) versus lower (40 mmol (1.6 g) K/day) K intake in adults with stage 3 CKD.

## 2. Materials and Methods

This trial was an investigator-initiated, single-center, double-masked, 2-period, crossover feeding trial sponsored by the American Heart Association that took place between July 2009 and June 2011. All participants provided written informed consent. This study was approved by the Institutional Review Board of Johns Hopkins University School of Medicine and was registered at clinicaltrials.gov (NCT00949585).

### 2.1. Participants

Eligible participants were >18 years old and had stage 3 CKD (estimated GFR of 30–59 mL/min/1.73 m^2^) [18], clinic or ambulatory SBP 120–159 mmHg, and DBP < 100 mmHg on no treatment or on a stable anti-hypertensive medication regimen. Major exclusion criteria were screening serum K ≥ 5 mmol/L or <3.5 mmol/L, history of hyperkalemia; insulin-requiring or uncontrolled (HbA1c > 8 %) diabetes; use of a K supplement; symptomatic heart disease or chronic disease(s) that might interfere with trial participation; body mass index > 40 kg/m^2^; >14 alcoholic drinks/week; and major food allergies or intolerances. Individuals on a diuretic were eligible, if the person was on a stable dose for at least two months prior to screening, was not hypokalemic or hyperkalemic at screening, and did not require K supplementation. See Supplementary Table S1 for a complete list of eligibility criteria. The primary recruitment strategy was targeted mailing of brochures to patients with stage 3 CKD who attended medical and nephrology clinics affiliated with Johns Hopkins.

### 2.2. Controlled Feeding

During a 1-week run-in period, eligible participants received meals from both arms of the trial. After run-in, participants were randomized to one of two computer-generated sequences: higher- then lower-K diet, or lower- then higher-K diet. Each feeding period lasted 4 weeks, with an interim 3–4 week washout period, during which time participants ate whatever they chose. Participants and investigators were masked to diet sequence. Table 1 displays the nutrient composition of the two study diets as well as the corresponding nutrient guidelines from KDOQI at the time of the trial.

The goal of 100 mmol K/day in the higher-K diet was chosen because it approximated the upper limit of intake that KDOQI recommended for individuals with stage 3–4 CKD, when the trial was designed and implemented [10]. The goal of 40 mmol K/day in the lower-K diet achieved a difference of 60 mmol K between diets, albeit at a level slightly below the lower level recommended for stage 3 CKD. In a large meta-analysis of trials supplementing K to lower BP in the general population [19], the K dose of the intervention was >60 mmol/day above the control condition in all but two trials. The higher-K diet was similar to the DASH diet, which emphasizes K-rich fruits and vegetables, low-fat dairy products; includes whole grains, poultry, fish and nuts; and is reduced in red meat, sweets, and sugar-containing beverages [20]. The lower-K diet contained fruits and vegetables that were low in K; otherwise, the diets were similar.

Other nutrient goals of the diets were based in part on KDOQI guidelines, and included 13% energy from protein, 50% energy from carbohydrates, and 37% energy from total fat (<10% energy from saturated fat). At the 2000 kcal level, the dietary goals were approximately 1 g phosphorous/day and 3.3 g (145 mmol) sodium/day. The daily sodium goal was slightly higher than the recommended KDOQI intake to minimize the potential beneficial effects on BP from a lower-Na intake while also providing a more ideal (lower) intake of Na.

The feeding protocol was similar to that used in prior trials [20,21]. Calorie intake was adjusted to maintain body weight throughout the trial. Seven menu cycles were developed for each diet at 3 calorie levels (1600, 2000, and 2400 kcals/day). Micronutrient levels were adjusted using the Linear Index Method [22]. See Supplementary Table S2 for the nutrient targets by calorie level. Representative meal samples from each day at each calorie level were sent to a laboratory (Silliker US, Chicago Heights, IL) for analysis.

During the feeding periods, participants were provided all meals for breakfast, lunch, and dinner. On three separate days/week, they consumed their main meals at the research center. All other weekday meals and all weekend meals were provided to participants to be consumed off-site. Participants were required to eat all the study foods given to them, and not to eat any non-study foods. Participants were allowed to drink < one serving/day of coffee, tea or diet soda, and <three servings/week of alcohol-containing beverages. Intake of water and beverages low in K and caffeine were unrestricted.

### 2.3. Measurements

Participants wore the Spacelabs 90207 ABP device for a 24 h period at baseline and during the fourth week of each feeding period; ABP were set to record BP every 30 min. The data were checked to confirm that at least 14 acceptable readings were obtained during the daytime. The timing of “waking” and “sleeping” was based on self-report.

Trained staff measured clinic BP using the Omron HEM-907 automatic blood pressure monitor. Pre-randomization BP was assessed at three screening visits and once during run-in, then weekly during each feeding period and the end of the washout period. Three consecutive measurements were performed at each visit and then averaged. Mean baseline clinic BP was the average of all readings at the three pre-randomization visits.

Participants had 24 h urine collections for electrolytes (measured using flame emission spectrophotometry) and creatinine (measured using Jaffe method) at screening, then at the end of each feeding period to assess adherence. Urine specimens were sent to Johns Hopkins Clinical Laboratories (Baltimore, MD, USA) for analysis. Serum chemistry panels (including serum K) were measured at screening, run-in, weeks 1, 2 and 4 of each feeding period, and during washout. Serum specimens were collected in a serum separator tube, centrifuged, and immediately shipped to Quest Diagnostics for analysis. If there was a value ≥ 5.5 mmol/L, blood was redrawn to check the serum K level within 1–3 days. Participants with confirmed hyperkalemia were withdrawn from the study. During analysis, GFR was estimated using the CKD-Epidemiology equation [23].

### 2.4. Analysis

The primary outcome was 24 h ambulatory SBP. Other outcomes were clinic SBP and DBP, 24 h ambulatory DBP, and daytime and nighttime ambulatory SBP and DBP. Serum K was the primary measure of safety; adherence measures were 24 h urinary K excretion, and 24 h urinary sodium excretion. For each outcome, we determined the mean difference between end-of-period measures on the lower- and higher-K feeding periods. In sensitivity analyses, we used just the last two of the three consecutive BP measurements at the clinic visits based on several studies demonstrating this is a more accurate method to assess BP [24,25]. In other sensitivity analyses, we used repeated measures analyses. Participants without assessments at the end of the 4-week feeding period were excluded from our primary analysis

We estimated that a total of 26 participants would provide 80% power at *p* < 0.05 (two-sided) to detect a difference between the two diets of 4 mmHg or more in SBP, a difference that also has clinical relevance [26]. All analyses were conducted with STATA version 14.0 (Stata Corporation, College Station, TX, USA). All comparisons were performed using generalized estimating equation regression models with a Huber and White robust variance estimator, which assumed an exchangeable working correlation matrix [27]. Statistical significance was *p* < 0.05 (two-tailed).

## 3. Results

### 3.1. Baseline Characteristics

**Figure 1:**
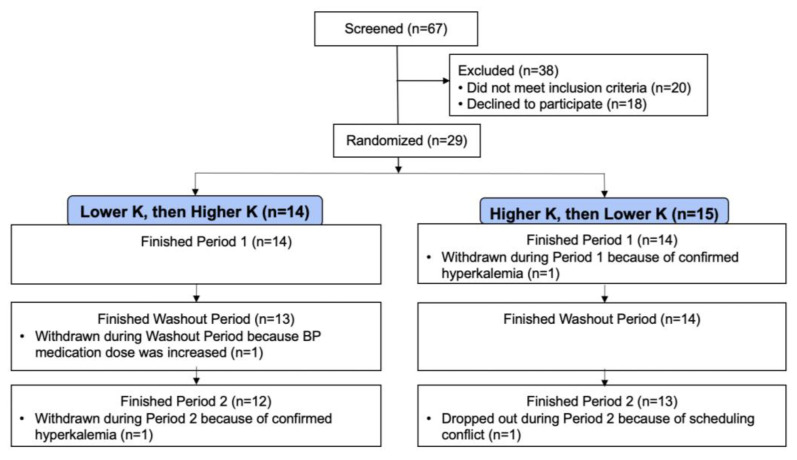
Consort diagram of the CKD-K trial.

Of 67 individuals who were screened in person, 29 individuals were randomized (Figure 1); 24 had ABP measurements at the end of both periods and 25 participants had 4-wk clinic BP measurements at the end of both study periods.

Among the 29 randomized participants, mean age was 67.2 years (SD, 11.6); 58.6% were female, and 69.0% were black (Table 2). At baseline, mean clinic SBP was 128.4 mm Hg (SD, 13.3), mean DBP was 71.3 mm Hg (SD, 7.8), mean serum creatinine was 1.3 mg/dL (SD, 0.2), and mean estimated GFR was 54.5 mL/min/1.73 m^2^ (SD, 11.7).

### 3.2. BP Results

Mean 24 h systolic ABP, the primary outcome, was 127.1 mmHg at baseline, 126.2 mmHg at the end of the lower-K period, and 124.1 mmHg at the end of the higher-K period (Table 3); the net difference (higher- minus lower-K period) was −2.12 mmHg [95% CI: −5.12, 0.87; *p* = 0.16]; the corresponding net difference in clinic systolic BP was greater, but not statistically significant: −4.21 mmHg [95% CI: −8.49, 0.07; *p* = 0.054]. While mean levels of all other BP measures were lower during the higher-K period compared to the lower-K period, none of the between-period differences were statistically significant.

### 3.3. Time Course of BP

**Figure 2:**
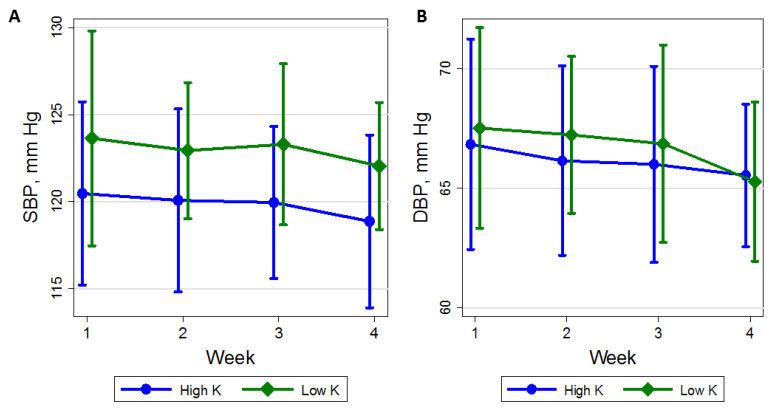
Mean (**A**) systolic blood pressure (mm Hg) and (**B**) diastolic blood pressure with 95% CI (vertical lines) for each week while on either the higher potassium (K) diet (circle) or the lower K diet (diamond). The number of participants contributing measurements by week (1–4) on the two diets were, 25, 25, 25, and 26 for the higher-K diet and 27, 26, 25, and 26 on the lower-K diet. SBP: systolic blood pressure; DBP: diastolic blood pressure.

The difference in mean clinic SBP between the higher- and lower-K diets was greater at week 4 (−4.21 mm Hg) than week 1 (−0.96 mm Hg); however, there was no significant trend in weekly change in SBP during either the higher-K diet (−0.64 mm Hg per week; 95% CI: −2.08, 0.80) or the lower-K diet (0.33 mm Hg per week; 95% CI: −1.19, 1.84; *P*-interaction was 0.34) (Figure 2; Supplementary Table S3). Similarly, the mean difference in DBP between the higher- and lower-K diets at week 1 was 0.81 mm Hg versus −0.08 mm Hg at week 4. The mean weekly change in DBP while on the higher-K diet was −0.53 mm Hg per week (95% CI: −1.36, 0.29) versus −0.32 mm Hg per week (95% CI: −1.10, 0.46) on the lower-K diet (*P*-interaction = 0.67).

### 3.4. Adherence Measures and Safety Monitoring

At 4 weeks on the lower-K diet, the mean 24 h urinary K was 39.9 mmol versus 81.4 mmol on the higher-K diet (Table 3), corresponding to a mean difference of 41.5 mmol (*p* < 0.001). Mean 24 h excretion of sodium was similar on both diets, i.e., 131.6 mmol on the lower-K diet and 118.5 mmol on the higher-K diet, corresponding to a mean difference of −13.1 mmol (*p* = 0.27).

**Figure 3:**
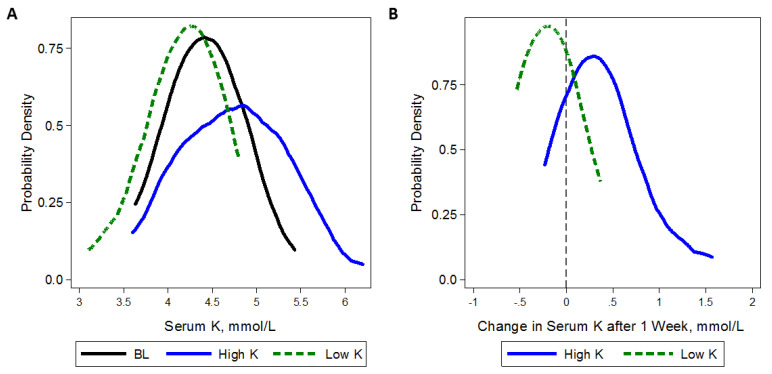
Kernel density plots of (**A**) serum potassium (K) levels (mmol/L) at baseline (BL) and after 1 week of higher (100 mmol/day, solid line) or lower (40 mmol/day, dash line) K intake; and (**B**) change in serum K levels after 1 week of higher (solid line) or lower (dash line) K intake. Baseline kernel density represents 3 measurements of serum K obtained after the Run-In visit, prior to starting the higher-K diet, and prior to starting the lower-K diet. In (**B**) gray vertical line represents a change of 0 mmol/L.

The higher-K diet shifted the distribution of serum K toward higher values. Mean serum K was 4.4 mmol/L at week 4 on the higher-K diet and 4.2 mmol/L at week 4 on the lower-K diet, with a statistically significant difference of 0.21 mmol/L (*p* = 0.003). The distribution of serum K at baseline, 1 week after the lower-K diet, and 1 week after the higher-K diet as well as the distribution of 1-week change in serum K from either diet is displayed in Figure 3.

Six participants had at least one hyperkalemic (serum K > 5.5 mmol/L) value during the higher- or lower-K periods; the odds of hyperkalemia on the higher- vs. lower-K diet during the trial was 2.50 (95% CI: 1.04, 6.00; *p* = 0.04, Table 4). Two of the 6 participants had a confirmed serum K value of >5.5 mmol/L (these occurred during the higher-K period), and both were withdrawn from the trial. One of the withdrawn participants was on tacrolimus and developed a serum K of 7.1 mmol/L; the pariticipant also had a rise in serum creatinine from a nadir of 1.37 mg/dL on the lower-K diet to 1.73 mg/dL when hyperkalemic on the higher-K diet. After the trial, the investigators became aware that this participant had a history of substantial hyperkalemia (an exclusion criterion). The other participant who developed confirmed hyperkalemia (5.7 mmol/L) had diabetes mellitus and was on both an angiotensin receptor blocker and an angiotensin-converting enzyme inhibitor.

Hypokalemia (defined as a serum K < 3.5 mmol/L) occurred in two participants. One participant had one hypokalemic value while in the washout period and one hypokalemic value on the low-K diet. The other participant had one hypokalemic value during the low-K period. Both participants were on hydrochlorothiazide (HCTZ), a K-wasting diuretic. One of these participants was also on triamterene (a K-sparing diuretic), and the other participant was also on an angiotensin converting enzyme inhibitor.

### 3.5. Sensitivity Analyses

We performed two sensitivity analyses in which we used just the last two of three BP measurements at each clinic visit and utilized a repeat measures approach that incorporated BP measurements from weeks 1, 2 and 3 during each period (Supplementary Table S4). In analyses using the last two of three BP measurements, there was a significant reduction in SBP at four weeks associated with the higher-K diet (mean net difference of −4.98 mmHg). In analyses incorporating repeated measurements, there was a significant reduction in SBP at weeks 3–4 weeks, whether or not all three BP measurements at each visit or just the last two of three BP measurements were used.

## 4. Discussion

In this randomized feeding trial of adults with stage 3 CKD, SBP 120–159 mmHg, and DBP < 100 mmHg, we documented that a higher dietary intake of K did not significantly lower systolic 24 h ABP, the primary outcome of the trial. The corresponding decrease in clinic SBP on the higher-K diet was of borderline statistical significance, and several sensitivity analyses suggest that the higher-K diet lowered BP. On the higher-K diet, serum K increased by an average of 0.21 mmol/dL, and two participants developed confirmed hyperkalemia (serum K > 5.5 mmol/L).

The effects dietary K intake in persons with CKD has not been well-studied. In a non-randomized, fixed-sequence pilot study, 11 individuals with stage 3 CKD were fed a baseline, control diet (2.4 g/ K/day) for one week and then the Dietary Approaches to Stop Hypertension (DASH) diet (4.7 g K/day) for two weeks [28]. In addition to its higher K content, the DASH diet also increases other nutrients including magnesium, calcium, fiber, protein, and calcium. There was no difference in clinic and mean 24 h ABP between baseline and DASH diets, but nighttime systolic BP was lower on the DASH diet. No hyperkalemic episodes were observed. However, the trial was only 2 weeks long and was a pre-post, non-randomized intervention study.

Two randomized trials documented beneficial effects on CKD from a diet rich in base-producing fruit and vegetables and therefore likely high in K. Goraya et al. randomized 108 patients with stage 3 CKD and serum bicarbonate 22–24 mmol/L to usual care, sodium bicarbonate, or base-producing fruits and vegetables. Preservation of estimated GFR and lower systolic BP (not primary outcomes) were noted after 3 years on the diet higher in fruits and vegetables [29]. The same authors reported in a study of 71 nondiabetic stage 4 CKD patients with metabolic acidosis that at one year, SBP was significantly lower than its respective baseline in the fruits and vegetables group but not the sodium bicarbonate group; one-year SBP was also significantly lower in the fruits and vegetables group than in the sodium bicarbonate group [30]. To our knowledge, no other trial has specifically examined the BP effects of specifically manipulating dietary K intake in individuals with stage 3 CKD.

We chose to study the effects of K intake in individuals with CKD because of evidence in animals [31] and in the general population, especially in individuals with hypertension [32], that a high K intake lowers BP. Furthermore, diets rich in K appear to reduce the risk of stroke and may be associated with decreased CVD mortality, via BP-dependent and BP-independent mechanisms [5,31,33,34,35,36,37]. Based on evidence from animal studies, mechanisms by which increased K might be protective include reduced oxygen free radical formation [38], renal inflammation [39], proliferation of vascular smooth muscle cells [40], endothelial dysfunction [41], macrophage adherence to the vascular wall [42], platelet aggregation [43], arterial thrombosis [44], and arterial stiffness [45].

In addition to the potential effects discussed above, animal and human studies have demonstrated that a high K diet may actually protect against kidney damage [3,4,5,6]. In a classic study, Tobian et al. documented that in sodium-loaded Dahl salt-sensitive rats, a diet containing either additional K citrate or K chloride resulted in a BP-independent slowing of the progression of kidney injury and prevented renal vascular, glomerular, and tubular damage [6]. Ellis et al. also showed that high K intake prevented hypertensive renal lesions in sodium-loaded spontaneously hypertensive rats, independent of BP [3]. Chronic K depletion has been shown to cause deterioration of kidney function, interstitial nephritis, or cyst formation in animals and humans [46,47,48,49,50,51,52].

Although diets high in K are recommended for the general population, patients with CKD are often told to limit dietary K due to concerns about hyperkalemia. Renal K excretion, however, may not significantly decrease until GFR is severely decreased (<10–20 mL/min/1.73 m^2^) [12,13,14]. Intestinal K excretion has also been shown in older studies to increase in CKD, which helps protect against substantial K retention [53]. Therefore, patients with moderate CKD may particularly benefit from higher rather than lower K intake, due to their high prevalence of hypertension and CVD, and because K may help to retard CKD progression [7,15,16]. In addition, many high-K foods, such as fruits and vegetables, have additional health benefits. Restricted K intake may also worsen constipation, which could lead to higher intestinal absorption of K, and may actually result in hyperkalemia [54]. The fact that limiting or avoiding many plant-based foods may contribute to adverse metabolic states (e.g., metabolic acidosis, oxidative stress, inflammation) and conditions (e.g., constipation and a rise in BP) has been proposed as a potential benefit of a higher-K diet that may possibly counteract its hyperkalemia-inducing effects [55,56].

A study in rats with CKD due to partial nephrectomy demonstrated that K supplementation suppressed renal inflammation, decreased the degree of interstitial injury, and lowered BP compared to rats on a low-K diet [39]. This could have considerable practical implications in humans as well. It has been postulated that some of the benefit of angiotensin converting enzyme inhibitors, angiotensin receptor blockers, and aldosterone antagonists may come from a rise in serum K (if it occurs), especially if baseline K is low. In individuals who do not develop a concerning degree of hyperkalemia, increased K intake may provide benefits. Of note, there were no hyperkalemic events in the studies of Goraya et al., who provided a diet rich in fruits and vegetables to individuals with stage 3 and stage 4 CKD who were on an angiotensin converting enzyme inhibitor (+/− hydrochlorothiazide in the stage 3 CKD study) [29,30]. Hyperkalemic events were also not reported in the pre-post study of the DASH diet in stage 3 CKD patients [28].

Despite the potential benefits of K, liberalizing dietary K in stage 3 CKD patients is not a common practice, especially in those who have high serum K values, have diabetes mellitus, or are on medication(s) that can raise serum K. In our study, six participants were found to have a serum K > 5.5 mmol/L, which was confirmed in two participants, both of whom were withdrawn from the study. One of these participants, who was on tacrolimus (which can raise serum K), had a previous history of repeated episodes of hyperkalemia that was unknown to the investigators until after the study; this participant should have been excluded. That participant also had a rise in serum creatinine concurrent with the hyperkalemia; hence, there may have been a degree of acute kidney injury leading to a decrease in urinary K excretion. The other withdrawn participant had diabetes mellitus and was on both an angiotensin converting enzyme inhibitor and an angiotensin receptor blocker. Of the other four participants who had hyperkalemia events that were *not* confirmed with a subsequent lab draw, three were on either an angiotensin converting enzyme inhibitor or angiotensin receptor blocker, and one was on tacrolimus. One of the four participants had diabetes mellitus.

There are several caveats related to interpretation of serum K levels in our trial. First, serum K values do not necessarily reflect intracellular K stores. Second, serum K measurements are highly variable and often inaccurate, with several well-known reasons for spurious elevations [57,58]. Serum K levels may also fluctuate substantially in patients with CKD, particularly in certain populations, such as those taking tacrolimus. Third, our relatively frequent assessments of serum K may have detected transient hyperkalemia that otherwise might not have been detected

Although the potential consequences of hyperkalemia are widely appreciated, more rapid CKD progression and a higher mortality in CKD patients have been reported in individuals who are hypokalemic and even in those with a low-normal serum K [59]. Both hyperkalemia and hypokalemia have been reported to be independently associated with an increased risk of untoward outcomes such as CVD, hospitalization, and all-cause mortality among patients with non-dialysis CKD [60,61]. In our trial, two participants were found to have hypokalemia on the lower-K diet. Both participants were on HCTZ in addition to a medication that can raise serum K. Therefore, limiting dietary K intake in individuals at risk for hypokalemia may lead to complications. Because of these concerns, the KDIGO recently modified its recommendations for dietary K intake in CKD patients towards a more individualized approach. Instead of providing a uniform recommendation for K intake, the group now recommends that “it is reasonable to adjust dietary K intake to maintain serum K within the normal range” [62].

Patiromer and sodium zirconium cyclosilicate bind potassium in the gut and decrease serum K levels. These medications could lead to a more liberalized K-rich diet as well as use of renin-angiotensin-aldosterone system (RAAS) inhibitors in patients with hyperkalemia. However, dietary K modification has not been studied in the setting of K binder use. Further research is needed to determine whether K binders in combination with a plant-based diet can improve RAAS-inhibitor use and reduce the risk for recurrent hyperkalemia among patients with CKD.

Our study has limitations. First, our sample size was small; however, as a cross-over trial, statistical power is substantially increased in comparison to a parallel arm trial with the same number of participants. Second, despite the broad inclusion criteria for BP, baseline 24 h ambulatory and clinic systolic BP was < 130 mmHg. It is well-recognized that for most nonpharmacologic and pharmacologic interventions, BP reductions are more substantial at higher levels of baseline BP [63]. With a larger sample size and higher BP, our trial would have had greater statistical power. Third, the feeding periods may have been too short to see a statistically significant reduction in BP. Lastly, the results of the study may not be generalizable to all individuals with stage 3 CKD; for example, we excluded certain populations, such as individuals who used insulin. However, we also included individuals with apparently normal serum K at baseline but who nonetheless were at a higher risk for hyperkalemia, such as persons with diabetes mellitus who were not taking insulin and transplant recipients on tacrolimus.

This study also has several strengths. Our trial incorporated a crossover, randomized design that allowed each participant to serve as their own control. Second, BP protocols were highly standardized by using trained observers who were masked to diet sequence. Third, we used ABP monitoring. Fourth, follow-up was high; only 4 participants did not complete both feeding periods. Fifth, overall adherence to the feeding protocol was excellent, as evidenced by urinary excretion of K and sodium. Sixth, the diets were designed to achieve a large contrast in potassium intake and to match other nutrients that affect BP.

## 5. Conclusions

In adults with stage 3 CKD, SBP 120–159 mm Hg, and DBP < 100 mm Hg, a higher dietary intake of K did not lower systolic 24 h ABP, the primary outcome of the trial. The corresponding decrease in clinic SBP with the higher-K diet was of borderline statistical significance. A higher dietary K intake increased serum K by an average of 0.21 mmol/L, and two at-risk participants developed confirmed hyperkalemia, defined as a serum K > 5.5 mmol/L; two participants developed hypokalemia on the lower-K diet. Additional research on the potential benefits and risks of increased dietary K is clearly warranted in the setting of CKD.

## Figures and Tables

**Table 1:** Study Diets Compared to National Kidney Foundation Guidelines.

	CKD-K Study Diet (Per 2000 kcal/day)		KDOQI Guidelines for Stage 3–4 CKD [10]
	Lower-K	Higher-K	
K, mg (mmol)	1600 mg (40 mmol)	3900 mg (100 mmol)	2000–4000 mg (51–102 mmol)
Na, mg (mmol)	3300 mg (143 mmol)	3300 mg (143 mmol)	<2400 mg (<104 mmol)
PO_4_, mg	1000	1000	800–1000
Protein, % kcal (g)	13% (65 g)	13% (65 g)	~10% (0.6–0.8 mg/kg)
Carbs, % kcal (g)	50% (250 g)	50% (250 g)	50–60
Sat Fat, % kcal	<10	<10	<10

KDOQI: Kidney Disease Outcomes Quality Initiative

**Table 2:** Baseline Characteristics ^‡^ of All Randomized Trial Participants (*N* = 29).

Age, years	67.2 (11.6)
Women, %	58.6
Black, %	69.0
Medications for:	
Diabetes	24.1%
Hypertension	93.1%
Specific types of medications	
ACEI or ARB	58.6%
K-sparing diuretic	13.8%
K-wasting diuretic	58.6%
Tacrolimus	6.9%
Body Mass Index, kg/m^2^	31.4 (4.7)
Mean clinic BP, mm Hg	
SBP	128.4 (13.0)
DBP	71.5 (7.7)
Serum creatinine, mg/dL	1.3 (0.2)
eGFR *, mL/min/1.73 m^2^	54.5 (11.7)

^‡^ Mean (SD) for continuous variables, and % for categorical variables. * CKD-EPI Equation.

**Table 3:** Effects of Higher- versus Lower- K Diet on Blood Pressure and Urine Electrolytes.

	*N*	Baseline	Lower-K	Higher-K	Difference (95% CI)) *	*p*-Value
**Systolic Blood Pressure (mmHg)**						
Clinic	25	121.5 (11.9)	122.8 (9.2)	118.6 (12.4)	−4.21 (−8.49, 0.07)	0.054
** 24 H Ambulatory	24	127.1 (14.5)	126.2 (12.2)	124.1 (11.7)	−2.12 (−5.12, 0.87)	0.16
Daytime Ambulatory	24	131.6 (14.5)	129.1 (11.4)	126.9 (10.5)	−2.26 (−5.44, 0.93)	0.17
Nighttime Ambulatory	24	118.5 (16.4)	121.0 (14.9)	118.4 (16.4)	−2.54 (−6.27, 1.19)	0.18
**Diastolic Blood Pressure (mmHg)**						
Clinic	25	67.3 (9.7)	65.2 (8.8)	65.1 (7.2)	−0.08 (−2.25, 2.09)	0.94
24 H Ambulatory	24	71.6 (9.1)	71.0 (7.8)	70.3 (7.0)	−0.70 (−2.46, 1.06)	0.44
Daytime Ambulatory	24	75.0 (9.4)	74.2 (7.9)	73.3 (7.5)	−0.97 (−2.90, 0.97)	0.33
Nighttime Ambulatory	24	65.0 (10.3)	65.6 (8.5)	65.0 (8.4)	−0.62 (−2.67, 1.43)	0.55
**Adherence Measures**						
Urine K, mmol/day	24	53.2 (23.7)	39.9 (16.2)	81.4 (33.6)	41.52 (28.38,54.66)	<0.001
Urine Na, mmol/day	24	128.8 (66.2)	131.6 (63.1)	118.5 (41.3)	−13.13 (−36.26,10.00)	0.27
Serum K, mmol/L	25	4.5 (0.5)	4.2 (0.4)	4.4 (0.4)	0.21 (0.07, 0.35)	0.003
Urine Cr, gm/day	24	1.1 (0.4)	1.2 (0.6)	1.1 (0.4)	−0.08 (−0.30,0.1)	0.46
Urine Vol, L/day	25	2.0 (1.0)	1.7 (0.5)	1.7 (0.7)	0.04 (−0.1,0.2)	0.66

* Values on higher minus values on lower. ** Primary outcome of trial.

**Table 4:** Instances of hyperkalemia (K > 5.5 mmol/L) during the trial according to high versus low potassium diet.

	Week 1			Week 2			Week 4			OR (95% CI) * *p*
	Total *N*	N with Hyperkalemia	%	Total *N*	N with Hyperkalemia	%	Total *N*	N with Hyperkalemia	%	
High Potassium	28	3	10.7	27	2	7.4	26	0	0.0	2.50 (1.04, 6.00) 0.04
Low Potassium	28	0	0.0	26	1	3.8	26	1	3.8	

* Generalized estimating equation model accounting for repeat, within-person measurements. Because a few participants did not finish the trial, not all 29 participants contributed data to each week in this analysis.

## Data Availability

Deidentified participant data and data dictionary will be available at https://archive.data.jhu.edu/ starting 1 year after publication of this article, contingent upon institutional review board approval.

## Supplementary Materials

The following are available online at https://www.mdpi.com/article/10.3390/nu13082678/s1, Table S1: Complete List of Trial Exclusions, Table S2: Nutrient Targets by Calorie Level, Table S3: Effects of Increasing Potassium on Blood Pressure by Week as well as Weekly Change from Baseline, Table S4: Effects of Increasing Potassium on Blood Pressure—Sensitivity Analyses using Last 2 Blood Pressure Measurements or Repeat Measures Analyses.

## Abbreviations

ABP	Ambulatory Blood Pressure
BMI	Body Mass Index
K	Potassium
SBP	Systolic Blood Pressure
DBP	Diastolic Blood Pressure
CKD	Chronic Kidney Disease
CI	Confidence Interval
