# Supplementary material for: Randomized Trial on the Effects of Dietary Potassium on Blood Pressure and Serum Potassium Levels in Adults with Chronic Kidney Disease
Source: Nutrients. 2021 Jul 31;13(8):2678. doi: 10.3390/nu13082678 (PMC8398615; doi:10.3390/nu13082678)
Supplement: Supplementary file 1 [file nutrients-13-02678-s001.zip › nutrients-1294212-supplementary.pdf]

Table S1. Complete List of Trial Exclusions.

**Major inclusion criteria**
• Age ≥ 18
• Estimated GFR between 30-59 mL/min/1.73m^2 by the 4-variable Modification of Diet in Renal Disease (MDRD) study equation
• Screening systolic BP 120-159 mmHg and diastolic BP < 100 mmHg
• Willingness to follow strict dietary rules for 13 weeks and to come to the clinical research unit each weekday for one meal during the two study periods
**Major exclusion criteria**
• Screening serum K ≥ 5 mmol/L (or history of hyperkalemia) or < 3.5 mmol/L
• Insulin-requiring or uncontrolled (HbA1C >8.0%) diabetes mellitus
• Use of K supplements
• Use of digoxin
• Cardiovascular event < 6 months ago, symptomatic ischemic heart disease, or congestive heart failure
• Body mass index > 40 kg/m^2
• Chronic disease(s) that might interfere with trial participation
• Pregnancy or lactation
• 14 or more alcoholic drinks/week
• Major food allergies
**Maintenance medications (allowed because we expect that individuals with this stage of CKD may commonly be on these medications)**
• Anti-hypertensive and lipid-lowering therapies were allowed if participants had been on a stable dose for at least two months
• Diuretics were allowed if participants had been on a stable dose for at least two months

Table S2. Nutrient Targets by Calorie Level.

	**Calorie level (kcal)**		
Nutrient	1600	2000	2400
Protein (g)	52	65	78
Carbohydrate (g)	200	250	300
Fat (g)	66	82	99
K (mg) (Lower, Higher)	1300/3600	1600/3900	1900/4200
Na (mg)	2640	3300	3960
Phosphorus (mg)	1000	1000	1000
Fiber (gm)	25+	25+	25+
Saturated Fat (% kcal)	8	8	8
Polyunsaturated Fat (% kcal)	12	12	12
Monounsaturated Fat (% kcal)	10	10	10

Table S3. Effects of Increasing Potassium on Blood Pressure by Week as well as Weekly Change from Baseline.

	Difference in mm Hg, High versus Low K (95% CI)							
	**N**	**Week 1**	**Week 2**	**Week 3**	**Week 4**	**Weekly Change - High K**	**Weekly Change - Low K**	**P-interaction**
Clinic SBP, mm Hg	25	−0.96 (-6.84, 4.92)	−2.51 (−7.78, 2.76)	−2.36 (−6.47, 1.75)	−4.21 (−8.49, 0.07)	−0.64 (−2.08, 0.80)	0.33 (−1.19, 1.84)	0.34
Clinic DBP, mm Hg	25	0.81 (−1.99, 3.61)	−0.68 (−3.23, 1.87)	−0.01 (−2.26, 2.24)	−0.08 (−2.25, 2.09)	−0.53 (−1.36, 0.29)	−0.32 −1.10, 0.46)	0.69

Table S4. Effects of Increasing Potassium on Blood Pressure – Sensitivity Analyses using Last 2 Blood Pressure Measurements or Repeat Measures Analyses (restricted to the subgroup with measurements each week, N=25).

		Difference in mmHg, High versus Low K (95% CI)						
		Each Week				Repeated Measures Analysis		
**All three measurements, mm Hg**	*N*	**Week 1**	**Week 2**	**Week 3**	**Week 4**	**Weeks 1-4**	**Weeks 2-4**	**Weeks 3-4**
Clinic SBP	25	−0.96 (−6.84, 4.92)	−2.51 (−7.78, 2.76)	−2.36 (−6.47, 1.75)	−4.21 (−8.49, 0.07)	−2.52 (−5.65, 0.62)	−3.02 (−6.08, 0.04)	**-3.24 (-6.43, -0.05)**
Clinic DBP	25	0.81 (−1.99, 3.61)	−0.68 (−3.23, 1.87)	−0.01 (−2.26, 2.24)	−0.08 (−2.25, 2.09)	−0.01 (−1.56, 1.55)	−0.27 (−1.82, 1.28)	−0.02 (−1.64, 1.59)
**Last two measurements, mmHg**								
Clinic SBP	25	0.22 (−5.64, 6.08)	−3.26 (−8.89, 2.37)	−2.45 (−6.98, 2.09)	**−4.98 (−9.41, −0.55)**	−2.60 (−5.83, 0.62)	**−3.54 (−6.83, −0.25)**	**−3.64 (−6.92, −0.36)**
Clinic DBP	25	0.88 (−2.02, 3.78)	−0.84 (−3.21, 1.53)	0.61 (−1.77, 2.99)	0.06 (−1.95, 2.07)	0.15 (−1.38, 1.69)	−0.08 (−1.60, 1.45)	0.35 (−1.20, 1.90)

Bold represents a $P < 0.05$.
